# The FomYjeF Protein Influences the Sporulation and Virulence of *Fusarium oxysporum* f. sp. *momordicae*

**DOI:** 10.3390/ijms24087260

**Published:** 2023-04-14

**Authors:** Chenxing Wei, Caiyi Wen, Yuanyuan Zhang, Hongyan Du, Rongrong Zhong, Zhengzhe Guan, Mengjiao Wang, Yanhong Qin, Fei Wang, Luyang Song, Ying Zhao

**Affiliations:** 1 College of Plant Protection, Henan Agricultural University, Zhengzhou 450046, China; 2 Institute of Plant Protection, Henan Academy of Agricultural Sciences, Zhengzhou 450002, China

**Keywords:** *Fusarium oxysporum*, YjeF protein, sporulation, virulence

## Abstract

*Fusarium oxysporum* causes vascular wilt in more than 100 plant species, resulting in massive economic losses. A deep understanding of the mechanisms of pathogenicity and symptom induction by this fungus is necessary to control crop wilt. The YjeF protein has been proven to function in cellular metabolism damage-repair in Escherichia coli and to play an important role in Edc3 (enhancer of the mRNA decapping 3) function in *Candida albicans*, but no studies have been reported on related functions in plant pathogenic fungi. In this work, we report how the *FomYjeF* gene in *F. oxysporum* f. sp. *momordicae* contributes to conidia production and virulence. The deletion of the *FomYjeF* gene displayed a highly improved capacity for macroconidia production, and it was shown to be involved in carbendazim’s associated stress pathway. Meanwhile, this gene caused a significant increase in virulence in bitter gourd plants with a higher disease severity index and enhanced the accumulation of glutathione peroxidase and the ability to degrade hydrogen peroxide in *F. oxysporum*. These findings reveal that FomYjeF affects virulence by influencing the amount of spore formation and the ROS (reactive oxygen species) pathway of *F. oxysporum* f. sp. *momordicae*. Taken together, our study shows that the *FomYjeF* gene affects sporulation, mycelial growth, pathogenicity, and ROS accumulation in *F. oxysporum*. The results of this study provide a novel insight into the function of *FomYjeF* participation in the pathogenicity of *F. oxysporum* f. sp. *momordicae*.

## 1. Introduction

*Fusarium oxysporum* is a serious soil-borne pathogenic fungus that is present around the world and that causes vascular wilt in more than 100 plant species. It causes destructive wilt disease in a number of cucurbitaceous plants, including bitter gourd, cucumbers, melons and watermelons, and so on. *F. oxysporum* f. sp. *momordicae* was first reported in 1982 [1] and has resulted in significant economic losses in the production of bitter gourd every year. Because *F. oxysporum* can survive in the soil for a long time, it is difficult to control, even when using effective chemicals.

*F. oxysporum*’s infection process in host plants can be divided into four stages: identification, spore germination and hyphal growth, colonization, and spreading. When the pathogen infects a host plant’s root cell, it first secretes signal substances (such as fusaric acid) to create immune reactions within the host plant, such as the generation of reactive oxygen species (ROS) and localized cell death (HR response), supporting the growth of the pathogen in the root [2]. After the hyphae enter the host’s root cell, conidia continue to be produced and to germinate between the root cells, and the hyphae begin to elongate and move along the vascular bundle [3]. As time goes by, the hyphae gradually penetrate the cell wall, colonize the host cell, and continue to grow and spread inside and between the host cells [3]. During this process, *F. oxysporum* produces secondary metabolites (such as toxins and cell-wall degrading enzymes) to break through the defense mechanisms of the host plant for further infection, resulting in the blockage of the vascular bundles of the host plant as well as leaf yellowing and wilting [4]. In the late stage of infection, the host plant will die, and the pathogen will return to the soil and seek out new hosts. Therefore, further understanding of *F. oxysporum* is required to develop efficient strategies to prevent this disease.

In recent years, understanding of the pathogenicity of *F. oxysporum* has seen huge advances [5]. The *Fosp9* gene has been reported as an essential factor for the virulence of *F. oxysporum* f. sp. *cubense* [6]. The *SGE1* gene plays a major role in the pathogenicity of *F. oxysporum* f. sp. *lycopersici* at the parasitic growth stage [7]. The SIX1 effector protein is secreted while *F. oxysporum* is colonizing its hosts and is required for virulence in *F. oxysporum* [8,9]. The serine proteases FoMep1 and FoSep1, secreted by *F. oxysporum* f. sp. *lycopersici*, have chitinase-cleaving activity and can significantly reduce the antifungal activity of tomato chitinase and reinforce the fungal virulence [10].

For soil-borne diseases, the spore of the pathogen is a key pathogenic factor for colonizing plant roots and infecting entire plants. Therefore, conidia production is closely related to pathogenicity. In *F. graminearum,* the *Type II myosin* gene is required for conidia production and virulence. The disruption of this gene can reduce conidiation by 76-fold and pathogenicity by 97% in wheat [11]. The *WOR1-like* gene plays a key role in pathogenicity and is involved in the developmental processes of conidia formation in *F. graminearum*. Without this gene, mutant strains show greatly reduced virulence, a low-level conidia count, and defects in spore development [12]. Some genes of *F. oxysporum* related to sporulation have also been reported. Fga1 encodes a Gα subunit protein that is involved in conserved signal transduction pathways, and the deletion of the *fga1*gene reduces the conidiation and pathogenicity of *F. oxysporum* [13]. Autophagy plays a vital role in regulating the morphology, cellular growth, and pathogenicity of *F. oxysporum.* Deleting *FoATG3* can result in a significant reduction in conidiation and virulence in potato tubers [14]. The *FoCPKA* knock-out strain has shown reduced spore attachment affinity to roots, lower spore production levels, and lost virulence [15]. In the absence of *FoMC69*, the mutant significantly reduces root rot symptoms, and its chlamydospores continue to increase during infection [3].

Reactive oxygen species (ROS) are a natural by-product formed by the metabolism of oxygen and play an important role in cellular immune response. ROS are considered a signal molecule of plant response to infection by pathogens. ROS signals are likely generated by the plasma membrane NADPH oxidase (Noxs) [16]. In fungi, there are three isoforms known as Noxs: NoxA, B, and C, which are essential for fungal infection structures. In *Verticillium dahlia*, the knocked-out *NoxA* mutants show significantly reduced virulence [17]. In *Alternaria alternata, NoxR* has been revealed to be required for the accumulation of hydrogen peroxide, and *NoxR* negatively regulates the expression of the *NoxA* gene [18].

The YjeF protein in *Escherichia coli* is an enzyme involved in cellular metabolism damage repair [19]. Meanwhile, YjeF has been identified as an enzymatic repair system that catalyzes the dehydration of NADHX and NADPX [19]. The current reports on YjeF mainly focus on its function as a domain of Edc3 (enhancer of the mRNA decapping 3). For example, Sbp1 (selenium-binding protein 1) has been reported to directly interact with Edc3 through the YjeF-N domain in yeast [20]. In *Candida albicans,* the YjeF domain plays a key role in the Edc3 function. When the YjeF domain is knocked out, the phenotypes change and ROS accumulation decreases [21]. This is the only reported function of YjeF in fungi. Although we have found that the *YjeF* gene is ubiquitous in plant pathogenic fungi, it has not been reported in pathogenic fungi of the YjeF protein function.

In previous studies, we found a strain carrying an ourmia-like virus, SD-V, with reduced pathogenicity [22]. The transcriptome analysis found that the expression of the *FomYjeF* gene of *F. oxysporum* f. sp. *momordicae* in the nonpathogenic strain was significantly up-regulated compared with the pathogenic strain. Therefore, we assume that the *FomYjeF* gene is related to the pathogenicity of *F. oxysporum*. In this study, we report on the *FomYjeF* gene in *F. oxysporum* f. sp. *momordicae*, which contributes to virulence by influencing the amount of spore formation and the ROS pathway. This study aims to explore the function of the *FomYjeF* gene in *F. oxysporum* and to analyze its effect on the pathogenicity of *F. oxysporum*, which may improve our understanding of the pathogenicity mechanisms in *F. oxysporum* and provide an important experimental basis for function research on the *FomYjeF* gene.

## 2. Results

### 2.1. Characterization and Phylogenetic Analysis of the FomYjeF Gene

**Figure 1:**
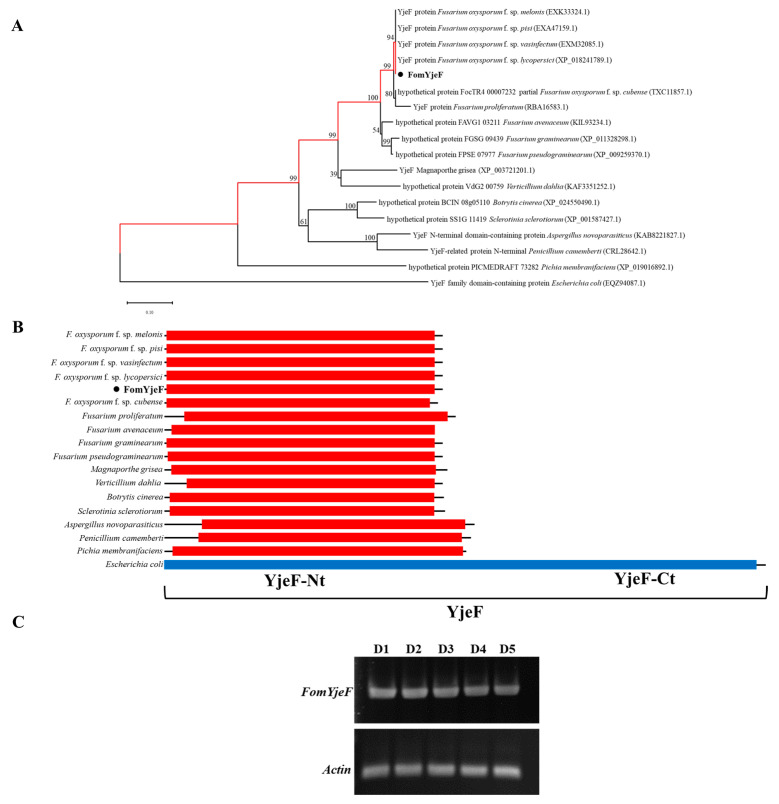
Sequence analysis of FomYjeF. (**A**) The phylogenetic tree was constructed based on amino acid sequences of FomYjeF and other fungi and bacteria using the neighbor-joining method with 1000 bootstrap replicates. The numbers at the nodes of the phylograms denote the bootstrap confidence values. (**B**) Phylogenetic analysis of conserved domains was structured based on the fungi and bacteria of the phylogenetic tree. (**C**) The everyday expression level of *FomYjeF* was determined by RT-PCR. *Actin* was a reference gene. D1–D5 represented the 5 day transcription level of *FomYje*F in SD-1. The FomYjeF-IN-F/R and Actin-F/R primer sets were used.

The gene sequence of *FomYjeF* contains 1268 bp with six introns and is predicted to encode a protein with 237 amino acids. A phylogenetic tree based on the amino acid sequence showed that the *FomYjeF* and homolog *YjeF* genes in *Fusarium* species are more closely related than other fungi (Figure 1A). Additionally, they contain similar domains (YejF-Nt) of fungi while being different from the structure in bacteria (Figure 1B). RT-RCR showed that the expression level of this gene is always the same (Figure 1C).

### 2.2. Effects of FomYjeF on Normal Mycelial Growth and Conidia Production

To determine the function of the *FomYjeF* gene, the knock-out mutant FomYjeF-KO-2 was generated in the wild-type (WT) strain of *F. oxysporum,* SD-1, via the targeted replacement of the *FomYjeF* gene with a hygromycin-resistance gene cassette (*HYG*). PCR assays with a gene-specific primer set revealed that the *FomYjeF* gene was replaced by the *HYG* cassette in FomYjeF-KO-2, and we also generated complement strains by introducing *FomYjeF* under the native promoter into one representative deletion strain, FomYjeF-CO-2 (Figure S1).

**Figure 2:**
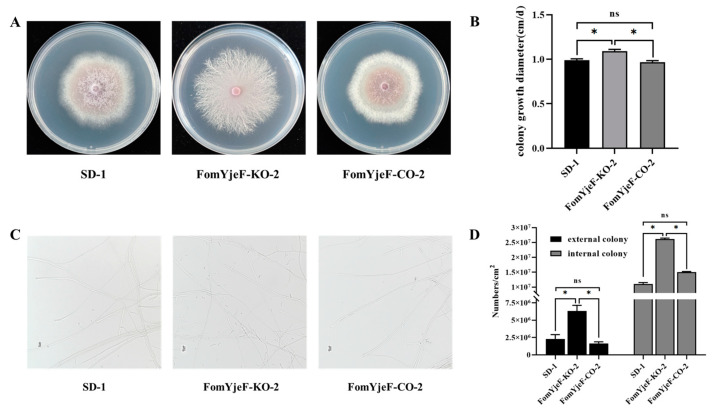
Vegetative growth, mycelial growth, and conidia production of wild-type SD-1, *FomYjeF* deletion, and *FomYjeF*-complemented strains. (**A**) Wild-type SD-1, FomYjeF-KO-2, and FomYjeF-CO-2 were cultured on PDA plates at 28 °C in dark for 5 days. (**B**) Growth rates. (**C**) Hyphae of wild-type SD-1 and mutant strains observed by EM. Bars:20µm. (**D**) Macroconidia production of wild-type SD-1 and all mutants were counted in per square centimeter after culturing on PDA for 4 days (See Figure S2 for the schematic diagram of sampling points). * over the histogram indicates standard error of the mean (SEM) at *p* < 0.05 (two-way ANOVA test). ns indicates no significant difference. Error bars represent the SEM of three biological replicates with three technical replicates.

The FomYjeF-KO-2 strain has significantly different mycelium in solid media compared with the WT strain SD-1 after growing on potato dextrose agar (PDA) plates at 28 °C for 5 d. The fomYjeF-KO-2 strain showed light purple or white colony phenotypes, while the SD-1 strain produced purple pigment. Meanwhile, the mycelium of the FomYjeF-KO-2 strain became sparse, similar to as if there was material accumulation, while it was restored in FomYjeF-CO-2 (Figure 2A). Moreover, the FomYjeF-KO-2 strain showed radial growth of 1.092 ± 0.066 cm/d, whereas WT strain SD-1 measured 0.989 ± 0.056 cm/d and the FomYjeF-CO-2 strain measured 0.969 ± 0.059 cm/d (Figure 2B). These results indicate that disruption of the *FomYjeF* gene might affect fungal mycelium growth. No obvious differences were observed among the FomYjeF-KO-2, FomYjeF-CO-2, and SD-1 strains in terms of the mycelial morphology on the PDA medium, whereas the deletion of mutant FomYjeF-KO-2 exhibited megasporogenesis formation (Figure 2C). Meanwhile, the macroconidia numbers of the FomYjeF-KO-2 strain were more significantly increased than they were in the SD-1 and FomYjeF-CO-2 strains during external and internal colony sampling, as shown Figure S2. After growing on PDA for 4 d, the FomYjeF-KO-2 strain obtained 6.3 ± 0.85 × 10^6^ macroconidia/cm^2^ for the external colony and 2.6 ± 0.05 × 10^7^ macroconidia/cm^2^ for the internal colony, in contrast to 2.3 ± 0.6 × 10^6^ macroconidia/cm^2^ for the external colony and 1.1 ± 0.07 × 10^7^ macroconidia/cm^2^ for the internal colony of the SD-1 strain and 1.7 ± 0.2 × 10^6^ macroconidia/cm^2^ for the external colony and 1.5 ± 0.03 × 10^7^ macroconidia/cm^2^ for the internal colony of the FomYjeF-CO-2 strain (Figure 2D). The results showed that the *FomYjeF* gene is involved in the negative regulation of the sporulation pathways in *F. oxysporum* and that the disruption can strongly improve the capacity of macroconidia production in *F. oxysporum*.

### 2.3. FomYjeF Regulates Responses to Abiotic Stress and Fungicides

To determine whether the *FomYjeF* gene engages in stresses such as adversity, growth rate inhibition between the mutant strain FomYjeF-KO-2 and the WT strain SD-1 was measured in different environments. Samples were grown on PDA plates with added 0.05% Congo red, 0.05% SDS, and 1.5 M sorbitol, respectively, at 28 °C for five days. There were no obvious differences in colony diameters, color, or mycelial growth and inhibition rate among mutant strain FomYjeF-KO-2, WT strain SD-1, and FomYjeF-CO-2.

**Figure 3:**
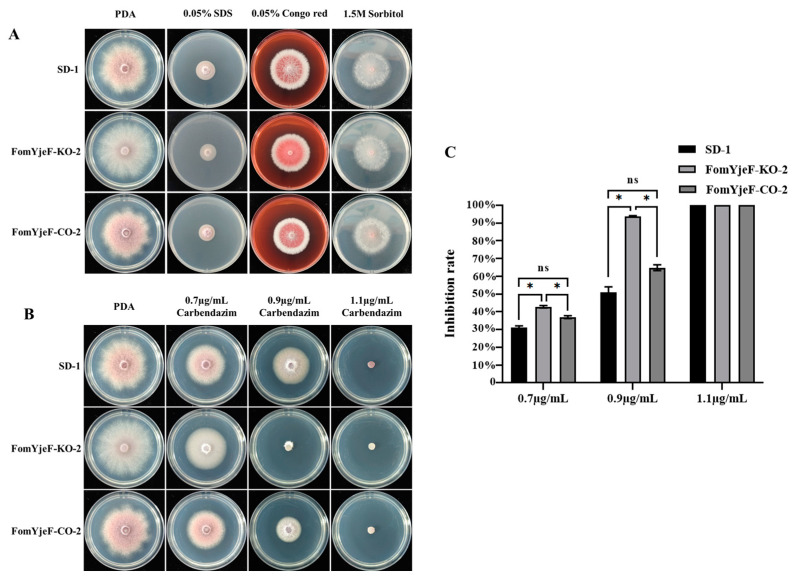
The abiotic stress and carbendazim response of mycelial growth in wild-type SD-1, *FomYjeF* deletion, and *FomYjeF*-complemented strains. (**A**) Colonies of SD-1, FomYjeF-KO-2, and FomYjeF-CO-2 were cultured on a PDA medium amended with 0.05% SDS, 0.05% Congo red, and 1.5 M sorbitol. (**B**) Colonies of SD-1, FomYjeF-KO-2, and FomYjeF-CO-2 were cultured on a PDA medium amended with 0.7 µg/mL, 0.9 µg/mL, and 1.1 µg/mL carbendazim. (**C**) The inhibition rate of mycelial growth of carbendazim. All Petri dishes were incubated at 28 °C for 5 days. * over the histogram indicates standard error of the mean (SEM) at *p* < 0.05 (two-way ANOVA test). ns indicates no significant difference. Error bars represent the SEM of three biological replicates with three technical replicates.

Carbendazim is a broad-spectrum fungicide. After being cultured on PDA plates with added carbendazim, the mutant strain FomYjeF-KO-2 barely grew on the 0.9 µg/mL carbendazim (inhibition rate: 94%), while the WT strain SD-1 and FomYjeF-CO-2 strain only showed inhibition rates of 51% and 65% (Figure 3B,C). The inhibition rate of the FomYjeF-KO-2 strain was also significantly higher than that in SD-1 and FomYjeF-CO-2 with 0.7 µg/mL carbendazim (Figure 3C). These results show that the *FomYjeF* gene is involved in the carbendazim-associated stress pathway and that they have little effect on the Congo red, SDS, and sorbitol-related stress pathways.

### 2.4. FomYjeF Contributes to the Pathogenicity of F. oxysporum

**Figure 4:**
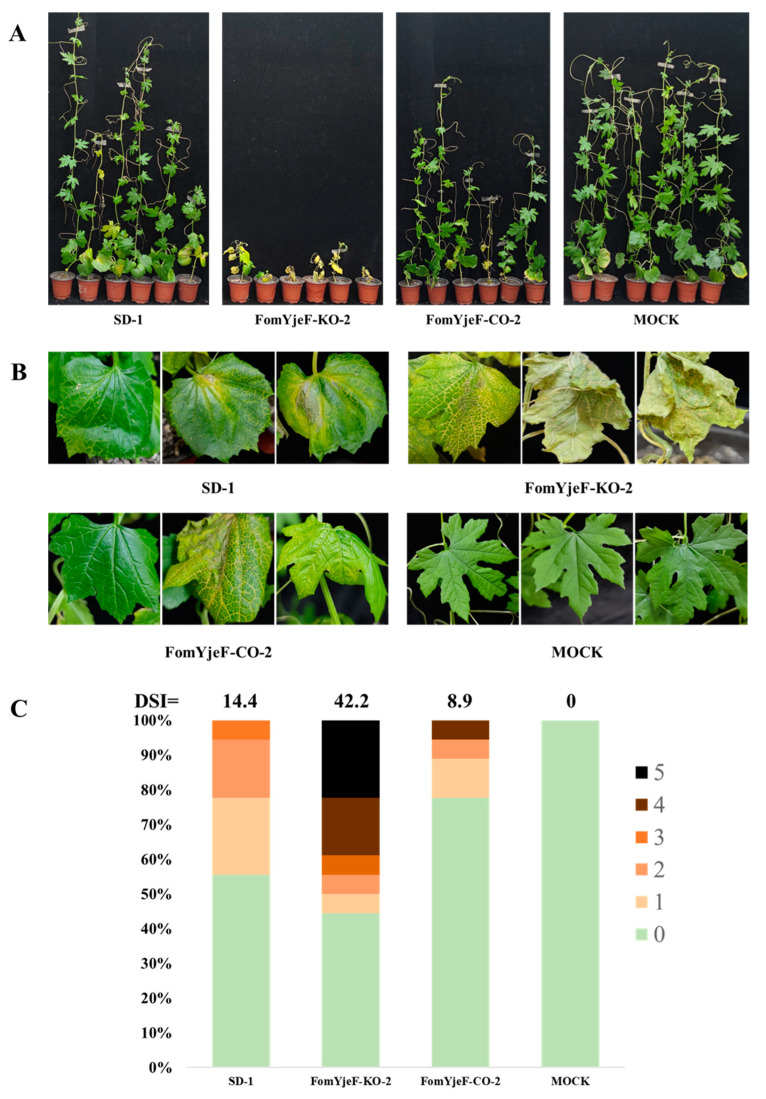
FomYjeF regulated the pathogenicity of *F. oxysporum* f. sp. *momordicae*. (**A**) The wild-type strain SD-1, FomYjeF-KO-2, and FomYjeF-CO-2 were inoculated to bitter gourd plantlets at the two-leaf stage for 28 days. As the control, MOCK was inoculated with sterile water. (**B**) Yellow symptoms in the leaf veins of bitter gourd. (**C**) Disease severity index (DSI) of bitter gourd plants inoculated with SD-1 and mutant strains. The disease severity was recorded using a scale ranging from 0 to 5, with 0 for healthy plants and 5 for dead plants.

The virulence of the *F. oxysporum* strains was evaluated on bitter gourd plantlets by root infection assays at the two-leaf stage. After 4 weeks of inoculation, the pathogenicity of the FomYjeF-KO-2 strain was significantly enhanced compared with that of the WT strain and FomYjeF-CO-2 strain (Figure 4A). The plants and leaves had obvious dwarfing and wilt symptoms after being inoculated with the FomYjeF-KO-2 strain, while only yellowing symptoms were observed after being inoculated with either the WT strain SD-1 or the complement strain FomYjeF-CO-2 (Figure 4A,B). In addition, the disease severity index (DSI) increased to up to 42.2 after inoculation with the FomYjeF-KO-2 strain, but was only 14.4 and 8.9 infected when inoculated with the WT strain and complement strain, respectively (Figure 4C). These data indicate that the deletion of the *FomYjeF* gene enhances the virulence of *F. oxysporum* on bitter gourd.

### 2.5. FomYjeF Participates in Regulating ROS Accumulation in F. oxysporum

**Figure 5:**
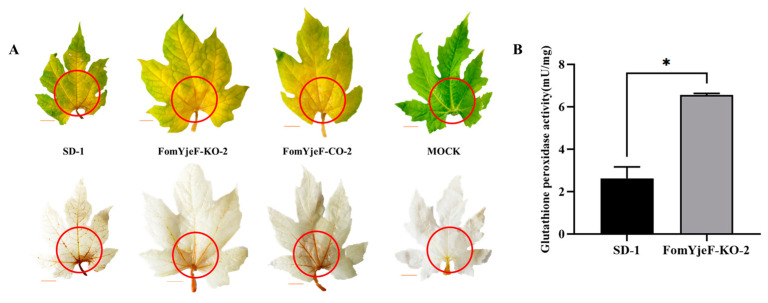
The deletion of *FomYjeF* affected the ROS accumulation and sensibility to oxidative stress of *Fusarium oxysporum* f. sp. *momordicae*. (**A**) The wild-type strain SD-1, FomYjeF-KO-2, and FomYjeF-CO-2 were inoculated to bitter gourd leaves of similar size and physiological state in the same part. MOCK leaf was inoculated with PDA. The red circle indicates the site of the disease and observation part. Bars: 1 cm. (**B**) Glutathione peroxidase activity in SD-1 and FomYjeF-KO-2. * over the histogram indicates the standard error of the mean (SEM) at *p* < 0.05 (*t*-test). Ns indicates no significant difference. Error bars represent the SEM of three biological replicates with three technical replicates.

ROS accumulation is an important indicator of plant response to pathogen infection, with H_2_O_2_ rapidly accumulating in and around the injection site. Meanwhile, H_2_O_2_ produces dark brown polymers when it encounters DAB (Diaminobenzidine). The DAB staining test was used to detect whether the *FomYjeF* gene participates in the ROS pathway in bitter gourd leaves. Seven days post-inoculation, the leaves inoculated with FomYjeF-KO-2 showed a larger lesion size but less H_2_O_2_ accumulation (dark brown polymer), in contrast to the leaves inoculated with WT strain SD-1 or the complement strain FomYjeF-CO-2. Leaves that were not inoculated showed no accumulation of H_2_O_2_ (Figure 5A). Moreover, we also measured the content of glutathione peroxidase, an enzyme that degrades peroxides such as H_2_O_2_, in the WT SD-1 and mutant FomYjeF-KO-2 strains. Three times more glutathione peroxidase was measured in the FomYjeF-KO-2 strain than in the WT SD-1 strain (Figure 5B). These results indicate that deletion of the *FomYjeF* gene enhances glutathione peroxidase accumulation and the ability to degrade the hydrogen peroxide in *F. oxysporum*.

### 2.6. FomYjeF Deletion Changes the Related Gene Expression of Sporulation and ROS Pathways

According to the above results, the deletion of the *FomYjeF* gene has an impact on the conidiation and pathogenicity of *F. oxysporum*. To verify whether *FomYjeF* gene deletion changes the expression of sporulation and ROS pathways in related genes or not, the expression levels of related genes were detected using qRT-PCR. The genes *Type II myosin* and *WOR1*, which are involved in spore production, were up-regulated three times and eight times more in the mutant FomYjeF-KO-2 strain than in the WT SD-1 strain, respectively. The gene *NoxR*, which is involved in the ROS pathway, was down-regulated in the mutant FomYjeF-KO-2 strain more than it was in the WT SD-1 strain, while the *NoxA* gene was up-regulated. This indicates that the deletion of the *FomYjeF* gene may be involved in the regulation of related pathways.

## 3. Discussion

In past studies, many genes have been reported to be related to the pathogenicity of *F. oxysporum*. For example, Fosp9 plays an essential role in the virulence of *F. oxysporum* [6]; *FoMC69* is required for pathogenicity and is an important factor of *F. oxysporum* infection [3]; the *ATG3* and *ATG22* genes are key regulators for the development and virulence of *F. oxysporum* [14,23]; and the *F. oxysporum* mutant strain loses the ability to infect its host without the *fmk1* gene [24]. The *YejF* gene is a gene that has been reported recently and that is involved in cellular metabolism damage repair by catalyzing the dehydration of NADHX and NADPX in *E. coli* [19]. It also plays a key role in the Edc3 function that is relevant to ROS accumulation [21]. However, the function of the *YjeF* gene in pathogenic fungi has not yet been reported. In a previous study, we found that the expression of the *FomYjeF* gene of *F. oxysporum* f. sp. *momordicae* was significantly up-regulated in nonpathogenic strains. The pathogenicity of the knock-out mutant strain was significantly enhanced (Figure 4A,B), which indicates that the *FomYjeF* gene is involved in the pathogenicity of *F. oxysporum.* This is the first study on the functional roles of *FomYjeF*, not only in *F. oxysporum*, but also in pathogenic fungi as well.

**Figure 6:**
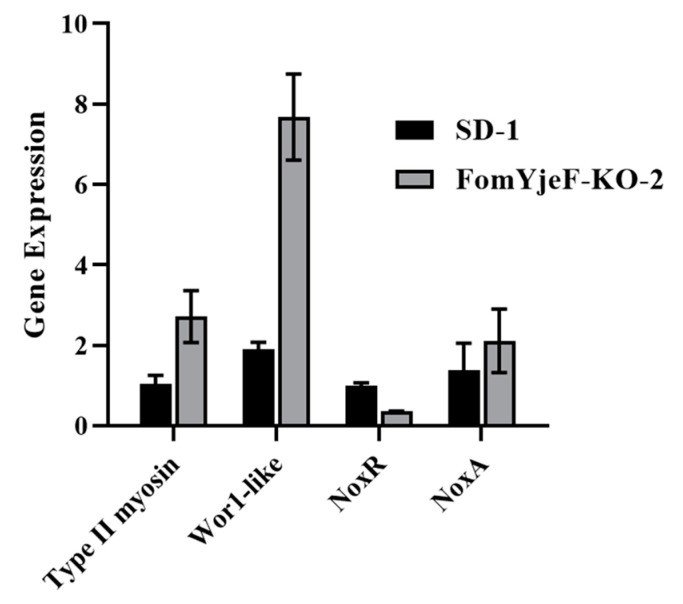
The transcription levels of *Type II myosin*, *Wor1-like*, *NoxR,* and *NoxA* in the wild-type SD-1 and FomYjeF-KO-2 according to q-PCR using the Type II myosin-F/R, Wor1-like -F/R, NoxR-F/R, and NoxA-F/R primer sets. Error bars represent the SEM of three biological replicates with three technical replicates.

For soil-borne pathogenic fungi, spores are a source of infection used to infect host plants. Therefore, the production and developmental processes of conidia are closely related to the pathogenicity of fungi. In previous studies, a number of pathogenic fungi genes have been reported to be involved in conidia formation or to affect conidia production, and they can also affect host pathogenicity. For instance, the disruption of the *Type II myosin* gene can significantly reduce the conidiation and pathogenicity of *F. graminearum* [11], and the *Wor1-like* gene also regulates the conidia formation and pathogenicity of *F. graminearum* [12]. In this study, we found that the internal and external conidia in the mutant strain, without the *FomYjeF* gene, were increased by 2.7 and 2.4 times compared with WT strain SD-1, respectively (Figure 2D). Meanwhile, the mutant strain FomYjeF-KO-2 showed a significantly enhanced pathogenicity, and the DSI increased three times (Figure 4). In previous studies, the gene deletion mutants of *F. oxysporum* such as *FoATG22*, *FoATG3, FoCPKA, FoMC69,* and so on, have been shown to reduce conidia production and virulence [3,14,15,23]. At the same time, the homologous genes in the *Type II myosin* gene and *Wor1-like* gene in *F. oxysporum* were up-regulated with the absence of the *FomYjeF* gene (Figure 6). In this study, pathogenicity increased as the conidia count increased, consistent with the above findings. This indicates that conidia production and conidia numbers are also closely related to the pathogenicity of *F. oxysporum*.

ROS plays an important role in cellular immune response, and ROS species are also used as the signaling molecules of plants in response to infection by pathogens [25]. *Noxs* genes are believe to regulate the ROS signals that are generated [14]. Past studies found that ROS are generated and accumulated at the infected site to prevent pathogen spread during pathogen infection [26,27]. To resist pathogen infection, the host initiated ETI, during which H_2_O_2_ rapidly accumulated in and around the injection site when *F. oxysporum* infected the bitter gourd plants. Meanwhile, H_2_O_2_ generates a dark brown polymer that is visible when it encounters DAB. There is also a certain correlation between the genes related to ROS production and the pathogenicity of fungi. For example, the *NoxA* gene regulates the virulence in *V. dahlia,* and the *NoxR* gene is required for ROS accumulation in *A. alternata* [17,18]. Therefore, we speculated that plants produce ROS to resist infection during *F. oxysporum* infection. In this work, we found that when the wild-type strain infected the bitter gourds, the leaves produced ROS and showed high levels of brown polymers after DAB staining; however, the knock-out mutant strain showed lower H_2_O_2_ accumulation and increased glutathione peroxidase activity at the same time (Figure 5). For this reason, we speculate that the absence of the *FomYjeF* gene caused less H_2_O_2_ accumulation that reduced the ETI of the plants, leading to further infection and increased pathogenicity. Moreover, the *NoxR* gene was down-regulated and the *NoxA* gene was up-regulated in the disruption mutant strain (Figure 6). Hence, we assume that *FomYejF* may regulate the pathogenicity through the ROS pathway.

In this study, we found that, without the *FomYjeF* gene, the knock-out mutant showed more sporulation, faster mycelial growth, more sensitivity to carbendazim, stronger pathogenicity, and lower ROS accumulation. This indicates that the deletion of *FomYjeF* affects conidia production and pathogenicity by acting on the spore production and ROS pathway. This is the first study on the functional roles of FomYjeF in pathogenic fungi. This work lays a foundation for *FomYjeF* gene function in pathogenic fungi and provides a further understanding of the pathogenic mechanism of *F. oxysporum.*

## 4. Materials and Methods

### 4.1. Fungal Strains, Host Plants, and Culture Conditions

The *F. oxysporum* wild-type strains SD-1 used in this study were *Fusarium oxysporum* f.sp. *momordicae* strains. All strains were cultured on potato dextrose agar (PDA; Difco Laboratories, Detroit, MI, USA) medium at 28 °C in the dark. In this study, we used PDA medium and PDB to test the mycelial growth rate and conidiation capacity of all strains, respectively. The host plant was bitter gourd Ruyu 33.

### 4.2. Analyses of Gene and Protein Sequences

The homologous proteins of FomYjeF were found using the BlastP program from the National Center for Biotechnology Information (NCBI) website (https://blast.ncbi.nlm.nih.gov/Blast.cgi, accessed on 23 May 2022.). The phylogenetic tree was generated by the neighbor-joining method using MEGA11 with 1000 bootstrap replicates using all homologous proteins of FomYjeF. Based on the phylogenetic tree, we searched all protein domains and motifs of the homologous proteins using the CD search program available on the NCBI website (https://www.ncbi.nlm.nih.gov/Structure/cdd/wrpsb.cgi, accessed on 6 June 2022).

### 4.3. Generation of FomYjeF Deletion and Complemented Mutants

The preparation of protoplasts and the transformation of SD-1 were carried out according to the polyethylene glycol-mediated transformation method [28]. Target gene deletion and replacement were generated according to the split-marker recombination (SMR) strategy [29]. For gene deletion, the 5′ and 3′ flanking of the *FomYjeF* gene were cloned from SD-1. Each amplicon and the hygromycin resistance cassette (*HYG*) were mixed and fused using PCR. The final product for transformation was generated during the third PCR step while using nested primer pairs. With transformation, the *FomYjeF* gene was replaced by the hygromycin resistance cassette. For the gene complement, we cloned a fragment containing the entire *FomYjeF* gene and its upstream sequence with the primers FomYjeF-CO-F and FomYjeF-CO-R and then cloned it into neoP3300-3. The complemented strain was then generated via agrobacterium-mediated transformation. The transformants were selected on plates with 100 µg mL^−1^ of hygromycin B or 100 µg mL^−1^ of G418. All primers are shown in Table S1.

### 4.4. Phenotypic Analysis of Mutants

To test the sensitivity of the strains to abiotic stress and fungicides, the PDA medium was amended with 0.05% sodium dodecyl sulfate (SDS), 0.05% Congo red, and 1.5 M sorbitol. For carbendazim, we set a gradient concentration. Three concentrations of carbendazim were set to test the stain responses to fungicides: 0.7 µg/mL carbendazim, 0.9 µg/mL carbendazim, and 1.1 µg/mL carbendazim.

### 4.5. Pathogenicity Assay of Mutants

To determine the pathogenicity of SD-1 and the mutants, 18 bitter gourd plantlets at the two-leaf stage were inoculated with each strain. A macroconidia mixture (2 × 10^6^ conidia/mL) was prepared as the inoculum. The test adopted the root dipping method, and each treatment was dipped in 40 mL inoculum for 20 min. At 28 days after inoculation, the incidence of all plants was calculated. The disease severity was recorded using a scale ranging from 0 to 5, with 0 for healthy plants and 5 for dead plants.

### 4.6. Detection of Intracellular Antioxidative Enzymes

The assay was performed using a Total Glutathione Peroxidase Assay Kit with NADPH (Beyotime Biotechnology; Shanghai, China). Mycelium was collected after being cultured on PDA at 28 °C in the dark for 3 days. Mycelium was lysed with the cell lysis buffer from the kit and then ground on ice. Then, the lysate was centrifuged at 12,000 r/min at 4 °C for 10 min, and the supernatant was used for the assay. In this assay, three blanks (no sample), three backgrounds (SD-1), and three samples were performed. The assay was repeated three times and was performed at 25 °C, 340 nm.

### 4.7. Method using 3, 3-Diamino-Benzidine Detecting H_2_O_2_ Production in Plant Leaves

To test H_2_O_2_ production in plant leaves, we chose leaves with the same growth size. Meanwhile, the SD-1 strain and mutants strain cake were inoculated at the petiole wound site and then kept moist in the light at 28 °C. After 7 dpi, in the late stage of the disease, the leaves were infiltrated with PH = 3.8, 1 mg/mL DAB solution and were left for 5 h at 25 °C in the dark. Afterward, the treated leaves were boiled in 95% ethanol to remove the chlorophyll.

### 4.8. Expression of Conidiation-Related and ROS-Related Genes

The mycelium and conidia mixtures of FomYjeF-KO-2 and wild-type SD-1 were cultured on PDA medium with cellophane. RNA was extracted from the mycelia and conidia described above using Trizol reagent (TransGen Biotech; Beijing, China). The mycelia were ground after freezing with liquid nitrogen, added to an enzyme-free centrifuge tube filled with 1 mL Trizol, left to stand at room temperature for 10 min, and centrifuged at 12,000 r/min for 15 min at 4 °C. The supernatant was aspirated, chloroform was added, and the supernatant was left to stand at room temperature for 10–15 min. After full layering, it was centrifuged at 12,000 r/min for 15 min at 4 °C. The supernatant was aspirated, the same volume of isopropanol was added, and the supernatant was left to stand at room temperature for 10 min before being centrifuged at 12,000 r/min for 15 min at 4 °C; then, the supernatant was removed, the precipitate was washed twice with 70% ethanol, DEPC-H_2_O was added to dissolve the precipitate, and the RNA sample was stored at −80 °C. cDNA was synthesized using a first-strand cDNA synthesis kit (Vazyme). During synthesis, 10 pg–5 µg RNA was added to an RNase-free centrifuge tube, RNase-free ddH_2_O to 8µL was added, and the mixture was heated at 65 °C for 5 min and then kept on ice for 2 min; then, 5 × gDNA wiper Mix was added, and the mixture was incubated at 42 °C for 2 min; 10 × RT Mix, HiScript III Enzyme Mix, Oligo (dT)_20_VN was added, and the mixture was left to incubate at 37 °C for 45 min. After incubation, the mixture was heated at 85 °C for 5 s to inactivate the enzyme, and the cDNA sample was stored at −80 °C. All gene data are shown in Table S1. Three biological replicates were performed.

## Data Availability

Data are contained within the article or Supplementary Material.

## Supplementary Materials

The supporting information can be downloaded at: https://www.mdpi.com/article/10.3390/ijms24087260/s1.
