# Supplementary material for: The FomYjeF Protein Influences the Sporulation and Virulence of Fusarium oxysporum f. sp. momordicae
Source: Int J Mol Sci. 2023 Apr 14;24(8):7260. doi: 10.3390/ijms24087260 (PMC10138616; doi:10.3390/ijms24087260)
Supplement: Supplementary file 1 [file ijms-24-07260-s001.zip › Supplementary file.pdf]

The FomYjeF Protein Influences Sporulation and Virulence of *Fusarium oxysporum* f. sp. *momordicae*

Chenxing Wei[1,†], Caiyi Wen[1,†], Yuanyuan Zhang[1], Hongyan Du[1], Rongrong Zhong[1], Zhengzhe Guan[1], Mengjiao Wang[1], Yanhong Qin[2], Fei Wang[2], Luyang Song[1], Ying Zhao[1*]

[1]College of Plant Protection, Henan Agricultural University, Zhengzhou 450046, China;

[2]Institute of Plant Protection, Henan Academy of Agricultural Sciences, Zhengzhou 450002, China

[†] These authors contributed equally to this work.

[*]Corresponding author

Ying Zhao

218 Pingan Avenue, Zhengdong New District, Zhengzhou, Henan Province, China 450046

E-mail: zhaoying@henau.edu.cn

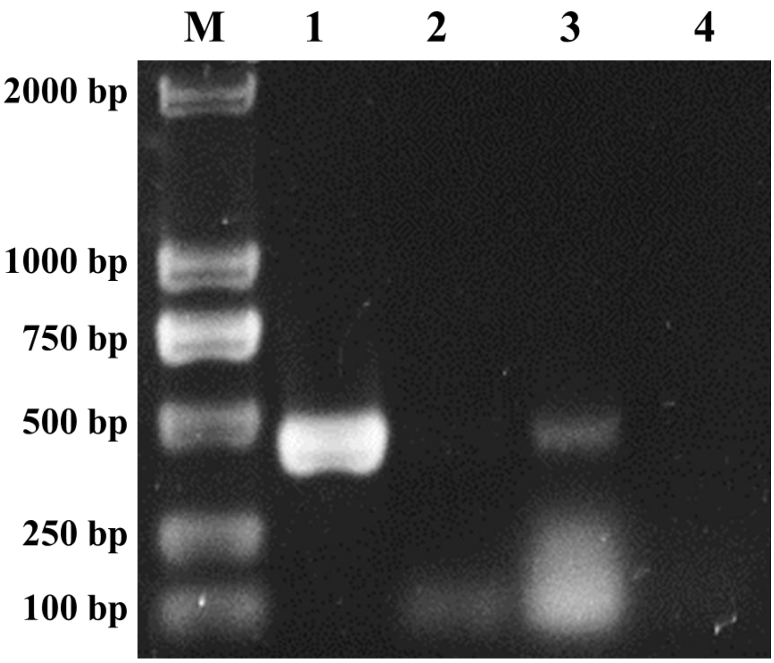

Figure S1. Confirmation of *FomYjeF* deletion and complemented by PCR. The FomYjeF-IN-F/R primer sets were used. Lane 1: wild-type SD-1; lane 2: FomYjeF-KO-2; lane 3: FomYjeF-CO-2; lane 4: MOCK (dd H_2O_2).

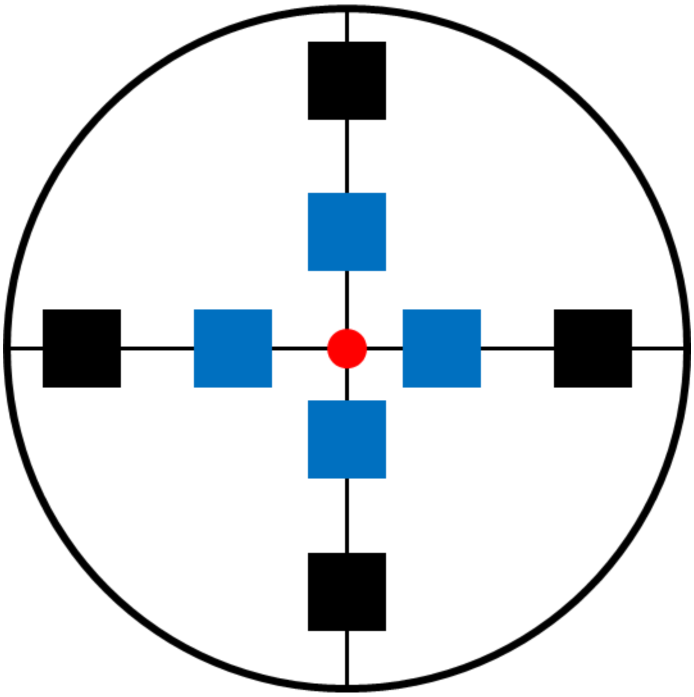

Figure S2. The schematic diagram of sampling points in Fig.2D. The black circle represents a petri dish and red circle represents a fungus cake. After culturing for 5 days, mycelia were sampled from the internal (blue square) and external (black square) of the petri dish. Each square measures one square centimeter.

Table S1. Primer sets used in this study.

Primer Names	Primer Sequence (5' to 3')
FomYjeF-S-F	TGACAAATGTTGGATACAGGCG
FomYjeF-S-R	GATCGCCATAATGCCTTTGAAT
FomYjeF-X-F	ACATGGTTGGAACGATTGCG
FomYjeF-X-R	ATCAGCGGCTCCGTTCAGT
FomYjeF-IN-F	AAGATTTCACTTCGGTCGCACA
FomYjeF-IN-F	CCATTCAACGATTCCCGCAG
HY-R	GTATTGACCGATTCCTTGCGGTCCGAA
YG-F	GGCTTGGCTGGAGCTAGTGGAGGTCAA
HYG-F	GGCTTGGCTGGAGCTAGTGGAGGTCAA
HYG-R	AACCCGCGGTCGGCATCTACTCTATTC
FomYjeF-CO-F	GGGGTACCCGTAAGGGTCGGGTTTAGGAAT
FomYjeF-CO-R	GGGGTACCTTTCTTCGGAATGGTATGCGTG
Actin-F	GGTAACCAAATCGGTGCTGCTTTC
Actin-R	ACCCTCAGTGTAGTGACCCTTGGC
Type II myosin-F	GCCATATTCTCCTCTCCTT
Type II myosin-R	AACCATGAGTTATTGCTGAT
Wor1-like-F	GGAACATGGTAATGAGTA
Wor1-like-R	ATGCTGATAGTCTTCTTG
NoxR-F	CATGATCGCTCCTGATAT
NoxR-R	ATGTCTTCGTCCTTAACC
NoxA-F	CTCAAGATTCACACCTAT
NoxA-R	TTGTTCGTGATTGTAGTT
